# Factors Associated With Smoking Cessation in Early and Late Pregnancy in the Smoking, Nicotine, and Pregnancy Trial: A Trial of Nicotine Replacement Therapy

**DOI:** 10.1093/ntr/ntt156

**Published:** 2013-10-14

**Authors:** Luis R. Vaz, Jo Leonardi-Bee, Paul Aveyard, Sue Cooper, Matthew Grainge, Tim Coleman

**Affiliations:** ^1^Division of Primary Care, University of Nottingham Medical School, Queen’s Medical Centre, Nottingham, UK;; ^2^Division of Epidemiology and Public Health, Nottingham City Hospital, University of Nottingham, Nottingham, UK;; ^3^Department of Primary Care Health Sciences, University of Oxford, Oxford, UK

## Abstract

**Introduction:**

Previous studies have found partners’ smoking status, multiparity, and nicotine dependence to be associated with smoking cessation in pregnancy. However, no studies have investigated influences on cessation among women using nicotine replacement therapy (NRT). We analyzed data from a trial of NRT in pregnancy to determine factors associated with shorter- and longer-term cessation.

**Methods:**

Data were collected at baseline, 1 month, and delivery from 1,050 pregnant women. Two multivariable logistic models for validated cessation at 1 month and delivery were created with a systematic strategy for selection of included factors.

**Results:**

All findings are from multivariable analyses. At 1 month, odds of cessation were greater among those who completed full time education at >16 years of age (odds ratio [*OR*] = 1.82, 95% confidence interval CI = 1.24–2.67, *p* = .002) but they were lower in women with higher baseline cotinine levels (*OR* = 0.93, 95% CI = 0.90–0.95, *p* < .001). At delivery, the odds of cessation were greater among those who completed full time education at >16 years of age (*OR* = 1.89, 95% CI = 1.16–3.07, *p* = 0.010) but were inversely associated with higher baseline cotinine levels (*OR* = 0.96, 95% CI = 0.92–0.99, *p* = .010).

**Conclusions:**

Women who are better educated and have lower pretreatment cotinine concentrations had higher odds of stopping smoking and factors associated with shorter and longer term cessation were similar.

## INTRODUCTION

Smoking in pregnancy is a significant public health problem. In the United Kingdom, a country with strong tobacco control culture, a survey in 2011 found that 26% of pregnant women smoked at some point before or during pregnancy and 12% smoked constantly throughout gestation (Eastwood, 2011). As smoking is a completely preventable cause of poor health outcomes for mothers and their babies, stopping smoking before or during pregnancy is vital. Unfortunately, though, there are few evidence-based cessation interventions that are proven to work for cessation in pregnancy.

A systematic review investigating the predictors of quit attempts made by nonpregnant smokers, found that a lower number of previous quit attempts and higher levels of nicotine dependence were both inversely associated with cessation (Vangeli, Stapleton, Smit, Borland, & West, 2011). Factors that have been associated with increased number of quit attempts in pregnancy also include age and smoking duration (Yu, Park, & Schwalberg, 2002). However, a recent systematic review found that having a partner who smoked, multiparity and increasing nicotine dependence had, in many studies, been found inversely associated with likelihood of achieving cessation (Schneider, Huy, Schutz, & Diehl, 2010). Additionally, socioeconomic factors such as increased income and educational levels of the mother and partner have also been shown to be associated with cessation in pregnancy (Ebert & Fahy, 2007; Mohsin & Bauman, 2005; Schneider et al., 2010), but these associations may be due to decline in smoking rates, which has been found to be lower in women from lower socioeconomic groups (US DHHS, 2004). Data from surveys conducted in the United Kingdom and Spain have also found that pregnant women with lower educational and socioeconomic levels have lower chances of cessation, whereas women who smoked fewer cigarettes, started smoking at an older age, had a partner who did not smoke or were primiparous were more likely to quit (Torrent et al., 2004).

There is less evidence, though, about which factors might influence women’s success when using nicotine replacement therapy (NRT) in cessation attempts made during pregnancy. Although nicotine dependence appears central to maintaining smoking behavior in pregnancy, attempts to promote cessation in pregnancy by addressing this with NRT have thus far been unsuccessful (Coleman, Chamberlain, Davey, Cooper, & Leonardi-Bee, 2012a). Further investigation of factors associated with cessation in pregnancy is warranted and analyses using data from studies in which an attempt has been made to treat nicotine dependence would be particularly informative. Recently, the Smoking, Nicotine, and Pregnancy (SNAP) trial, a large trial investigating the use of NRT for smoking cessation in pregnancy was conducted (Coleman et al., 2012b), and using the cohort of participants from this trial, we investigate independent associations between participants’ baseline characteristics and cessation at both early and late follow-up points to help ascertain whether or not any might be potential determinants of successful cessation.

## METHODS

### Data Source

Data for explanatory variables in these analyses were collected at baseline and outcome variable data were collected at two subsequent follow-up points within the SNAP trial (Coleman et al., 2012b). Trial participants were aged 16–45 years; of 12–24 weeks gestation; smoked ≥10 cigarettes prior to pregnancy and smoked ≥5 cigarettes currently; and had exhaled carbon monoxide (CO) readings of >8 parts per million (ppm).

### Treatment Protocol

Between May 2007 and February 2010, 1,050 participants were recruited to the trial from seven English hospital antenatal clinics. Research midwives collected baseline data, prescribed trial patches and provided face-to-face behavioral support at enrollment, and collected follow-up data at contacts; 1 month and delivery. Women received a behavioral support session lasting up to 1hr at enrollment. A quit date was also set within 2 weeks of enrollment and the follow-up points were measured from this. Women were offered additional behavioral support from the local National Health Service (NHS) stop smoking services throughout the trial to all participants according to the national standards, and research midwives provided telephone support when women were contacted on their quit date, 3 days after this and at 1 month. Participants were randomized to receive either NRT (15mg/16hr) or identical placebo patches. The first 4 weeks supply of patches was issued on the quit date, with a second batch of 4 weeks of patches given to those women reported not smoking and who had CO validation at the 1-month follow-up. Full methods (Coleman et al., 2012b) including the initial (Coleman et al., 2007) and final (Coleman et al., 2009) protocols for this study are published elsewhere.

### Baseline Data: Explanatory Variables

Prior to randomization, the following data were collected from participants: date of birth, ethnicity, age on completion of full time education, partner’s smoking status, parity, gestational age, body mass index, and previous use of NRT during their current pregnancy. Saliva and blood samples were taken for cotinine estimation, along with exhaled CO readings to estimate smoke and nicotine intake, respectively. Trial recruitment site and participants’ treatment assignment (i.e., NRT or placebo) were also available from the trial database.

### Outcome Variables

For analyses in this paper, we used validated cessation at 1-month postquit date and at delivery as outcome variables. At 1 month, cessation was defined as continuous abstinence from quit date to 1 month, validated by an exhaled CO reading of ≤8 ppm; and, at delivery, cessation was defined as continuous abstinence from a quit date until delivery, validated by an exhaled CO reading of ≤8 ppm and/or a saliva cotinine level of <10ng/ml. Participants who were lost to follow-up were coded as continuing smokers.

### Analysis Strategy

This analysis investigated associations between baseline characteristics of participants and cessation at 1 month after initiating treatment (i.e., from quit date) and at delivery. Two multivariable logistic models were built. Initially, for both models, variables were identified which had significant univariate associations (*p* ≤ .05) with validated cessation at each timepoint. Secondly, these variables were all entered into a multivariable model using stepwise backwards elimination to remove variables found to have nonsignificant associations with outcome (*p* > .05). Finally, variables which showed no association at the univariate level were entered into the models individually, to determine if they were subsequently associated with validated cessation. Treatment assignment was included as an *a-priori* confounder.

To maximize the number of participants included in the analysis, where possible, a missing category was created for categorical variables with missing data and imputation was planned for continuous variables (baseline cotinine) where >10% of cases had missing data. Where all missing values for an exposure occurred among people in the same outcome category (i.e., continuing smokers) or a small percentage of data was missing for a continuous variable, a univariate sensitivity analysis comparing all participants against those with complete data was conducted, to verify that they did not differ in their baseline characteristics.

All analyses were conducted using Stata 11.2 (College Station, TX).

## RESULTS

In the 1 month and delivery multivariate analyses, missing data for the categorical variable “age full time education finished” could not be included as all those with missing data were smokers at follow-up and inclusion as an extra category would perfectly predict the outcome. Furthermore, imputation for the continuous variable “baseline cotinine level” was not carried out due to data being missing for only 80 participants (7.6%). As a result, analysis was undertaken on 957/1,050 participants (91.1%), for whom complete exposure data were available. Including the 93 participants for whom some baseline data were missing in the final multivariable model, did not alter the results. An analysis based on achieving validated cessation was conducted and characteristics of the women included are detailed in Table 1. At 1 month 167 (17.5%), and at delivery 84 (8.8%) of the participants achieved validated cessation.

**Table 1.** Participant Characteristics for Cessation at 1 Month and Delivery

Variable	One month		Delivery	
	Smoking (*n* = 790)	Abstinent (*n* = 167)	Smoking (*n* = 873)	Abstinent (*n* = 84)
Age (median [IQR]) (years)	25 (21–30)	25 (21–31)	25 (21–30)	25 (21–31)
Primiparous	36.8%	37.1%	36.3%	42.9%
Age full-time education finished (years)				
≤16	80.5%	68.9%	79.6%	66.7%
>16	19.5%	31.1%	20.4%	33.3%
Ethnicity				
British: White	97.3%	96.4%	97.4%	95.2%
Other	2.7%	3.6%	2.6%	4.8%
Gestational age (median [IQR]) (weeks)	15 (13–18)	15 (13–18)	15 (13–18)	15 (13–20)
Baseline cotinine (median [IQR]) (ng × 10^−1^/ml)	12.7 (8.7–18.2)	9.1 (5.8–13.8)	12.4 (8.1–17.8)	10.6 (6.0–15.3)

*Note.* IQR = interquartile range.

### Factors Associated With Smoking Cessation at 1 Month

At 1 month, in the univariate analysis, women who finished full time education when they were >16 years had greater odds of cessation; women who had a higher baseline cotinine levels, and those who were recruited from trial site 4, were found to have lower odds of cessation (Table 2). The final multivariable model shows which baseline variables were independently associated with validated cessation at 1 month. Women who were aged >16 years when they finished full time education (odds ratio [*OR*] = 1.82, 95% confidence interval [CI] = 1.24–2.67, *p* = .002) had significantly increased odds of achieving validated cessation. Participants who had a higher baseline cotinine (*OR* = 0.94, 95% CI = 0.91–0.96, *p* < .001 for a 10ng/ml increase) had significantly lower odds of cessation at 1 month after quit date. The effect of trial recruitment site 4 did not remain significant when added to the multivariable model (*OR* = 0.69, 95% CI = 0.36–1.34, *p* = .277).

**Table 2.** Univariate and Multivariate Associations With Validated Cessation at 1 Month

Variable	*N*	Smoking at 1 month (%)	Cessation at 1 month (%)	Crude *OR*	95% CI		*p* value	Adjusted *OR*	95% CI		LRT *p* value
					Lower	Upper			Lower	Upper	
Age (years)^†^											
<20	147	129 (87.8)	18 (12.2)	0.64	0.36	1.12	0.250*	–	–	–	–
20–24	322	264 (82.0)	58 (18.0)	1.00	–	–		–	–	–	
25–29	228	183 (80.3)	45 (19.7)	1.12	0.73	1.73		–	–	–	
30–34	158	134 (84.8)	24 (15.2)	0.82	0.49	1.37		–	–	–	
35–39	78	63 (80.8)	15 (19.2)	1.08	0.58	2.04		–	–	–	
≥40	24	17 (70.8)	7 (29.2)	1.87	0.74	4.73		–	–	–	
Ethnicity											
Other	27	21 (77.8)	6 (22.2)	1.00	–	–	0.521	–	–	–	–
British: White	930	769 (82.7)	161 (17.3)	0.73	0.29	1.84		–	–	–	
Age full time education finished											
≤16	751	636 (84.7)	115 (15.3)	1.00	–	–	0.001	1.00	–	–	0.002
>16	206	154 (74.8)	52 (25.2)	1.87	1.29	2.71		1.82	1.24	2.67	
Partner smokes											
No	225	186 (82.7)	39 (17.3)	1.00	–	–	0.412	–	–	–	–
Yes	654	544 (83.2)	110 (16.8)	0.96	0.65	1.44		–	–	–	
Not applicable	78	60 (76.9)	18 (23.1)	1.43	0.76	2.69		–	–	–	
Parity											
≤1	649	535 (82.4)	114 (17.6)	1.00	–	–	0.919*	–	–	–	
2 or 3	256	211 (82.4)	45 (17.6)	1.00	0.68	1.46		–	–	–	
≥4	52	44 (84.6)	8 (15.4)	0.85	0.39	1.86		–	–	-	
Baseline cotinine (ng × 10^−1^/ml)											
12.1 (7.8–17.6), median (IQR)	957	790 (82.6)	167 (17.4)	0.93	0.90	0.95	<0.001	0.93	0.90	0.95	<0.001
BMI											
<18.5	28	27 (96.4)	1 (3.6)	0.18	0.02	1.34	0.987*	–	–	–	–
18.5–24.9	362	300 (82.9)	62 (17.1)	1.00	–	–		–	–	–	
25–29.9	267	213 (79.8)	54 (20.2)	1.23	0.82	1.84		–	–	–	
>30	254	212 (83.5)	42 (16.5)	0.96	0.62	1.47		–	–	–	
Missing	46	38 (82.6)	8 (17.4)	1.02	0.45	2.29		–	–	–	
Length of first behavioral support session											
16–30	143	123 (86.0)	20 (14.0)	1.00	–	–	0.478	–	–	–	–
31–45	791	648 (81.9)	143 (18.1)	1.36	0.82	2.25		–	–	–	
>60	23	19 (82.6)	4 (17.4)	1.29	0.40	4.20		–	–	–	
Previous preterm births											
0	872	718 (82.3)	154 (17.7)	1.00	–	–	0.577	–	–	–	–
≥1	85	72 (84.7)	13 (15.3)	0.84	0.45	1.56		–	–	–	
Use of NRT since pregnancy began											
No	914	756 (82.7)	158 (17.3)	1.00	–	–	0.548	–	–	–	–
Yes	43	34 (79.1)	9 (20.9)	1.27	0.60	2.69		–	–	–	
Trial recruitment site											
Site 1	102	81 (79.4)	21 (20.6)	1.00	–	–	0.032	–	–	–	–
Site 2	113	97 (85.8)	16 (14.2)	0.64	0.31	1.30		–	–	–	
Site 3	188	151 (80.3)	37 (19.7)	0.95	0.52	1.72		–	–	–	
Site 4	238	212 (89.1)	26 (10.9)	0.47	0.25	0.89		–	–	–	
Site 5	165	127 (77.0)	38 (23.0)	1.15	0.63	2.11		–	–	–	
Site 6	77	63 (81.8)	14 (18.2)	0.86	0.40	1.82		–	–	–	
Site 7	74	59 (79.7)	15 (20.3)	0.98	0.47	2.06		–	–	–	
Gestational age (weeks)											
12–19	766	637 (83.2)	129 (16.8)	1.00	–	–	0.326	–	–	–	–
20–24	191	153 (80.1)	38 (19.9)	1.23	0.82	1.83		–	–	–	

*Note.* BMI = body mass index; CI = confidence interval; IQR = interquartile range; LRT = likelihood ratio test; NRT = nicotine replacement therapy; *OR* = odds ratio.

*p value: test for trend.

^†^Age included as continuous variable in multivariate model to derive *p* value, equivalent to test for trend adjusting for covariates.

### Factors Associated With Smoking Cessation at Delivery

Table 3 shows the univariable and multivariable associations with validated cessation at delivery; the univariable results found women who finished full time education at >16 years and had lower baseline cotinine levels had increased odds of cessation and those with higher baseline cotinine levels had lower odds of cessation. In the final multivariable model, women who continued school beyond the compulsory minimum age (16 years) were more likely to stop smoking (*OR* = 1.89, 95% CI = 1.16–3.07, *p* = .010) and women with a higher baseline cotinine level were less likely to achieve cessation (*OR* = 0.96, 95% CI = 0.92–0.99, *p* < .010).

**Table 3.** Univariate and Multivariate Associations With Validated Cessation at Delivery

Variable	*N*	Smoking at delivery (%)	Cessation at delivery (%)	Crude *OR*	95% CI		*p* value	Adjusted *OR*	95% CI		LRT *p* value
					Lower	Upper			Lower	Upper	
Age											
<20	147	135 (91.8)	12 (8.2)	0.93	0.46	1.89	0.403*	–	–	–	–
20–24	322	294 (91.3)	28 (8.7)	1.00	–	–		–	–	–	
25–29	228	206 (90.4)	22 (9.7)	1.12	0.62	2.02		–	–	–	
30–34	158	145 (91.8)	13 (8.2)	0.94	0.47	1.87		–	–	–	
35–39	78	74 (94.9)	4 (5.1)	0.57	0.19	1.67		–	–	–	
≥40	24	19 (79.2)	5 (20.8)	2.76	0.96	7.96		–	–	–	
Ethnicity											
Other	27	23 (85.2)	4 (14.8)	1.00	–	–	0.300	–	–	–	–
British - White	930	850 (91.4)	80 (8.6)	0.54	0.18	1.60		–	–	–	
Age full time education finished											
≤16	751	695 (92.5)	56 (7.5)	1.00	–	–	0.009	1.00	–	–	0.010
>16	206	178 (86.4)	28 (13.6)	1.95	1.20	3.16		1.89	1.16	3.07	
Partner smokes											
No	78	199 (88.4)	26 (11.6)	1.00	–	–	0.193	–	–	–	–
Yes	225	604 (92.4)	50 (7.6)	0.63	0.38	1.04		–	–	–	
Not applicable	654	70 (89.7)	8 (10.3)	0.87	0.38	2.02		–	–	–	
Parity											
≤1	649	588 (90.6)	61 (9.4)	1.00	–	–	0.304*	–	–	–	–
2 or 3	256	235 (91.8)	21 (8.2)	0.86	0.51	1.45		–	–	–	
≥4	52	50 (96.2)	2 (3.8)	0.39	0.09	1.62		–	–	–	
Baseline cotinine (ng × 10^−1^/ml)											
12.1 (7.8–17.6), median (IQR)	957	873 (91.2)	84 (8.8)	0.95	0.92	0.98	0.002	0.96	0.92	0.99	0.010
Length of first behavioral support session											
16–30	143	135 (94.4)	8 (5.6)	1.00	–	–	0.258	–	–	–	–
31–45	791	718 (90.8)	73 (9.2)	1.72	0.81	3.64		–	–	–	
>60	23	20 (87.0)	3 (13.0)	2.53	0.62	10.34		–	–	–	
BMI											
<18.5	28	27 (96.4)	1 (3.6)	0.44	0.06	3.37	0.273*	–	–	–	–
18.5–24.9	362	334 (92.3)	28 (7.7)	1.00	–	–		–	–	–	
25–29.9	267	243 (91.0)	24 (9.0)	1.18	0.67	2.08		–	–	–	
>30	254	225 (88.6)	29 (11.4)	1.54	0.89	2.65		–	–	–	
Missing	46	44 (95.7)	2 (4.3)	0.54	0.12	2.35		–	–	–	
Previous preterm births											
0	872	795 (91.2)	77 (8.8)	1.00	–	–	0.852	–	–	–	–
≥1	85	78 (91.8)	7 (8.2)	0.93	0.41	2.08		–	–	–	
Use of NRT since pregnancy began											
No	914	837 (91.6)	77 (8.4)	1.00	–	–	0.106	–	–	–	–
Yes	43	36 (83.7)	7 (16.3)	2.11	0.91	4.91		–	–	–	
Trial recruitment site											
Site 1	102	90 (88.2)	12 (11.8)	1.00	–	–	0.397	–	–	–	–
Site 2	113	106 (93.8)	7 (6.2)	0.50	0.19	1.31		–	–	–	
Site 3	188	172 (91.5)	16 (8.5)	0.70	0.32	1.54		–	–	–	
Site 4	238	221 (92.9)	17 (7.1)	0.58	0.26	1.26		–	–	–	
Site 5	165	152 (92.1)	13 (7.9)	0.64	0.28	1.47		–	–	–	
Site 6	77	69 (89.6)	8 (10.4)	0.87	0.34	2.24		–	–	–	
Site 7	74	63 (85.1)	11 (14.9)	1.31	0.54	3.15		–	–	–	
Gestational age (weeks)											
12–19	766	704 (91.9)	62 (8.1)	1.00	–	–	0.147	–	–	–	–
20–24	191	169 (88.5)	22 (11.5)	1.48	0.88	2.47		–	–	–	

*Note.* BMI = body mass index; CI = confidence interval; IQR = interquartile range; LRT = likelihood ratio test; NRT = nicotine replacement therapy; *OR* = odds ratio.

*p value: test for trend.

## DISCUSSION

### Main Findings

We found that, among participants in a trial of transdermal nicotine patches in pregnancy, smoking cessation of 1-month duration and also until delivery were positively associated with finishing full time education beyond the compulsory age of 16 years and negatively associated with baseline cotinine levels. Leaving school at 16 years is a marker of social disadvantage and also an indicator of lower socioeconomic status, which is associated with decreased probability of quitting.

### Strengths and Limitations

The main limitation of this study was that a relatively restricted variety of variables were collected in the trial; in particular, there were few behavioral or socioeconomic measures, which, in some studies have been shown to influence cessation (Schneider et al., 2010). It also remains possible that differences in cessation rates observed in early and late pregnancy might be explained by unmeasured factors. Furthermore, as a number of significance tests were performed, some of the observed associations may have occurred by chance (i.e., Type I errors). However, as this is the first analysis employing multivariable methods to determine factors which were independently associated with smoking cessation in a trial of NRT patches, findings remain interesting. Additionally, our study sample was large and was mostly complete, permitting inclusion of 91% of trial participants in analyses. This will have increased the likelihood that weak associations between baseline factors and validated cessation in the trial database could be discovered. A final advantage of investigating predictors in a trial of NRT is that biochemically validated cessation was used; accuracy of self-reported cessation in pregnancy is typically low due to the perceived social acceptability of smoking during pregnancy.

### Findings in the Context of Previous Work

Two previous systematic reviews, in nonpregnant (Vangeli et al., 2011) and pregnant smokers (Schneider et al., 2010), respectively, have investigated predictors of smoking cessation. Both found that nicotine dependence is an important predictor of quit attempt success, with higher levels reducing the likelihood of a successful quit attempt. Our analyses provide complementary data showing, in the context of an NRT trial, that lower cotinine concentration increased the odds of cessation among trial participants in both early and late pregnancy. Cotinine levels are a marker of tobacco smoke exposure rather than being a measure of nicotine dependence. However, within these trial participants both plasma and saliva measures of cotinine have been found to be highly correlated with a validated measure of nicotine dependence in pregnancy (Kwok, Taggar, Cooper, Lewis, & Coleman, 2013), so it is likely that, lower levels of nicotine dependence, would also increase the likelihood of women in this study quitting too.

The previous reviews also investigated associations between socioeconomic factors and smoking cessation and found strong evidence for a positive association between higher socioeconomic status or, higher income levels and cessation in pregnancy (Schneider et al., 2010); but little evidence was found for an association between income, level of education or employment status, and cessation in nonpregnant smokers (Vangeli et al., 2011). The present study concurs with the previous findings that higher levels of social disadvantage are associated with worse outcomes in pregnant women who attempted cessation as part of an NRT trial.

In the previous review that investigated pregnant smokers’ quit attempts, all included studies used self-reported cessation measures (Schneider et al., 2010), which may be prone to underreporting of smoking behavior due to social desirability bias and, so, the validity of findings from empirical studies included in this review could be questioned. As nonsmokers are unlikely to report themselves as smokers, misclassification of women currently smoking as nonsmokers could bias the strength of associations between factors relating to smoking behavior and addiction and those measuring social disadvantage and cessation toward the null. In our dataset, misclassification of smoking status due to such biases in self-reported data cannot have occurred; consequently, our demonstration of the importance of nicotine dependence and social disadvantage to smoking cessation is likely to be valid.

Only one previous report has used validated cessation data to investigate factors associated with successful cessation in pregnancy (Fish et al., 2009). This analysis used data from the U.S. “Baby Steps” trial (Pollak et al., 2007) and found that women who were primiparous and who used more NRT were more likely to report cessation at 38 weeks gestation (Fish et al., 2009); however, the study sample was small (104 women) and only univariable associations were reported, so these findings are difficult to interpret. Our much larger study has greater power and used a multivariable analysis, which investigated the independent associations with cessation.

The analyses presented in this paper suggest that, in pregnant women who use NRT to attempt cessation, higher levels of social disadvantage and higher pretreatment cotinine levels are associated with worse cessation outcomes. It is possible that both associations are causal, though a mechanism for cotinine concentration affecting cessation through its contribution to nicotine dependence is more immediately obvious. Outside of pregnancy, nicotine dependence is also more easily remedied, for example, by NRT, which has been shown to reduce the strength of craving and be effective (Stead, Perera, Mant, & Lancaster, 2008). These findings have research implications; further work investigating how socioeconomic status may influence success in quit attempts could uncover factors that are amenable to intervention. Similarly, further research into the treatment of nicotine dependence may be indicated; there is currently no evidence that NRT is effective in pregnancy (Coleman et al., 2012a), but future studies using higher doses of nicotine than those which have been trialed could be undertaken. Nicotine metabolism is faster in pregnancy (Dempsey, Jacob, & Benowitz, 2002) and the standard doses of NRT that have been investigated may be too low to be effective. The finding that increasing cotinine concentration, which is strongly correlated with nicotine dependence (Kwok et al., 2013), is strongly associated with cessation failure should provide a spur to investigate this possibility.

### Conclusions and Recommendations

Among pregnant participants in a trial of NRT for smoking cessation, women who were not educated beyond the compulsory age for finishing school and those who had higher pretreatment cotinine concentrations were less likely to stop smoking throughout pregnancy. Women with these characteristics may require a different kind of smoking cessation support in pregnancy than others; however, it is not possible, from our data, to determine the nature of support that these women would find most helpful.

## FUNDING

This work was supported by a grant from the National Institute for Health Research (NIHR) Health Technology Assessment (HTA) Programme (HTA 06/07/01). Luis R. Vaz’s studentship, provided by the UK Centre for Tobacco Control Studies (UKCTCS), is funded by the UK Clinical Research Collaboration (UKCRC).

## DECLARATION OF INTERESTS

*TC, JL-B, and PA are members of the UKCTCS, a UKCRC Public Health Research: Centre of Excellence. TC, SC, and PA are members of the NIHR School for Primary Care Research. There are no potential conflicts of interest relevant to this study.*
